# The Use of Carbolized Catgut Ligatures

**Published:** 1872-03

**Authors:** 


### The Use of Carbolized Catgut Ligatures

Dr. George Buchanan reports in *The Practitioner* for July, 1871, a case of diffused traumatic aneurism upon which he had operated by laying open the sac and applying a ligature both above and and below the wound in the artery. Carbolized catgut ligatures were used, because it was thought they would produce obliteration of the artery without ulceration through its coats. Considerable discharge took place, but from first to last not a trace of decomposition or putrification could be observed. The most careful examination of the discharge failed to detect any appearance of the catgut ligatures, and they were probably retained and and imbedded in the tissues, occlusion of the vessels taking place without ulceration of the coats of the artery and discharge of the ligature. The patient made an excellent recovery.—*Philadelphia Medical Times.*

